# Physicians’ Social Skills – Conceptualization, Taxonomy, and Behavioral Assessment

**DOI:** 10.5334/pme.1171

**Published:** 2024-12-26

**Authors:** Simon M. Breil, Dorothee Amelung, Sebastian Oberst, Torsten Rollinger, Helmut Ahrens, Amelie Garbe, Martina Kadmon, Bernhard Marschall, Mitja D. Back, Harm Peters

**Affiliations:** 1 Department of Psychology, University of Münster, Germany; 2 Medical Faculty, Heidelberg University, Germany; 3 Dieter Scheffner Center for Medical Education and Educational Research, Dean’s Office for Study Affairs, Charité – Universitätsmedizin Berlin, Germany; 4 Institute of Education and Student Affairs, Faculty of Medicine, University of Münster, Germany; 5 Medical Education Sciences, DEMEDA, Faculty of Medicine, University of Augsburg, Germany; 6 JICE, Joint Institute for Individualisation in a Changing Environment, University of Münster and Bielefeld University, Germany

## Abstract

Social skills (e.g., assertiveness, empathy, ability to accept criticism) are essential for the medical profession and therefore also for the selection and development of medical students. However, the term “social skills” is understood differently in different contexts. There is no agreed upon taxonomy for classifying physicians’ social skills, and skills with the same meaning often have different names. This conceptual ambiguity presents a hurdle to cross-context communication and to the development of methods to assess social skills. Drawing from behavioral psychology, we aim to contribute to a better understanding of social skills in the medical context. To this end, we introduce a theoretically and empirically informed taxonomy that can be used to integrate the large number of different social skills. We consider how skills manifest at the behavioral level to ensure that we focus only on skills that are actually observable, distinguishable, and measurable. Here, behavioral research has shown that three overarching skill dimensions can be seen in interpersonal situations and are clearly distinguishable from each other: *agency skill* (i.e., getting ahead in social situations), *communion skill* (i.e., getting along in social situations), and *interpersonal resilience* (i.e., staying calm in social situations). We show that almost all social skills relevant for physicians fit into this structure. The approach presented allows redundant descriptions to be combined under three clearly distinguishable and behavior-based dimensions of social skills. This approach has implications for the assessment of social skills in both the selection and development of students.

Physicians vary in how successfully they handle complex interpersonal situations, such as dealing with distressed patients, resistance from colleagues, or criticism from relatives. These individual differences in *social skills* relate to various positive outcomes [1,2] and are therefore part of all global competency frameworks for physicians (e.g., *communicator* and *leader* in CanMEDS, [3]; *interpersonal and communication skills* and *professionalism* in the Accreditation Council for Graduate Medical Education’s Outcome Project, [4]). Furthermore, years of research have highlighted the importance of social skills in selection (i.e., aiming to select applicants with high levels of specific social skills [5,6,7,8,9]) and development (i.e., facilitating students to develop specific social skills as part of the medical curriculum [10,11,12,13]).

Social skills are essential for future physicians, but ambiguities and idiosyncrasies plague their conceptualization and assessment in research and practice. That is, the term “social skills” is understood differently in different contexts. This ambiguity is due to there being no consensus taxonomy for the classification of physicians’ social skills, and many skills have been defined by an intuitive or experience-based (What are theoretically desirable social skills?) rather than empirical process (What differences in skills actually manifest in physicians’ behavior?; see also [14]). Furthermore, specific social skills with the same implied meaning are often described by different terms (e.g., location A refers to some measured skill as *empathy*, location B as *helpfulness*, but both locations refer to the same or very similar observed behavioral differences). Conversely, the same terms are often used to describe skills with different implied meanings (by *communication*, location A refers to comprehensibility of language, whereas location B refers to aspects of active listening; see also [15]). Taken together, these factors represent important challenges for cross-context communication and for the selection of methods that reliably assess and develop physicians’ social skills.

While there are reviews and commentaries on physicians’ general skills and desired competencies and their assessment [14,16,17,18,19,20], the role of social skills and their behavioral manifestation in medicine have not been discussed in depth. However, the assessment and conceptualization of social skills has been a focus of behavioral psychology for many years [2,15,21,22,23,24,25,26]. In this article, we seek to draw on key insights from behavioral psychology and apply these ideas and concepts to the study of physicians’ social skills. To this end, we (a) define the term “social skills,” (b) combine the relevant social skills of physicians into an integrated, empirically validated, and practically useful behavioral-based taxonomy, and (c) discuss how this taxonomy has implications for the assessment of social skills.

## Conceptualization and Definition of Physicians’ Social Skills

Historically, different approaches have been applied to conceptualize skills (or competencies)^1^ in different disciplines [23,25,26,28,29]. For example, skills have sometimes been characterized as abilities to solve *specific* situational requirements (i.e., according to this understanding, a person’s level of skill would depend solely on the specific situation in which they are observed). Recent research has moved towards a more cross-situational understanding of skills: A clear and empirically validated classification and assessment of different social skills is achieved only through an understanding of skills that does not depend on specific situations. Otherwise, it would be necessary to determine specific (socially) competent or (socially) incompetent behavioral patterns for every conceivable situation. Such an approach would be particularly impractical for the assessment of skills in the context of selection and development, where the aim is to select individuals who will be able to meet future requirements (selection) or to prepare individuals for future requirements (development). For this objective, a cross-situational understanding of skills is more suitable.

However, a cross-situational understanding of skills does not imply that individuals will always behave in the same way across all situations and contexts. Rather, skills can be seen as potentials to show certain behaviors, and thus refer to behavioral capacities that promote effective functioning in relevant situations (see [23,25,26] for similar definitions). This follows a modern, transactional perspective in which behavioral expression is the result of a complex interplay between person characteristics (for example the capacities to show specific behaviors) and environmental variables [30,31,32,33]). In the case of *social* skills, these capacities revolve around behaviors that are beneficial in *interpersonal* situations. Thus:

*Social skills refer to the entire range of skills (behavioral capacities) that promote effective functioning in interpersonal situations*.

In the context of medical practice, these situations include not only interactions with patients, but also with relatives, superiors, inferiors, and interdisciplinary and interprofessional teams. Social skills – often used synonymously with the terms interpersonal, soft, or people skills [25,27] – can be distinguished from more cognitive skills (e.g., reasoning) and more intrapersonal skills (e.g., time management, goal regulation).

Differences between individuals in specific social skills become visible in relevant interpersonal situations in which demonstrating these skills is *essential* (e.g., when only assertive behavior can effectively persuade someone). Individuals with high levels of specific social skills can recognize that specific behaviors are beneficial in certain situations and are able to act accordingly (e.g., assertive physicians recognize the situations in which assertive behavior is required and can implement it). By observing specific behaviors across multiple relevant situations, conclusions can be drawn about a person’s level of social skills [15,23].

**Figure 1:**
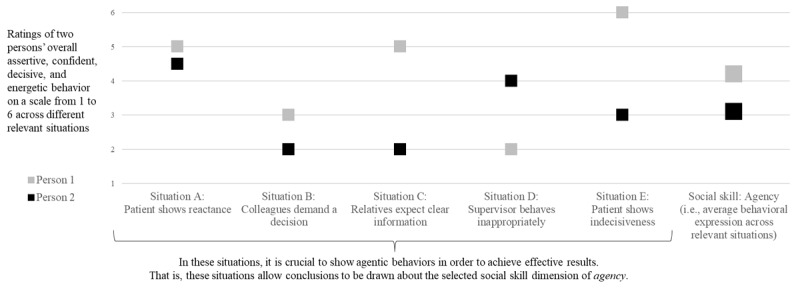
Conceptulization of Social Skills: The Interaction of Person Characteristics, Situations, and Behavioral Expression. Note. The hypothetical behavior of two individuals is shown. Person 1 has a higher level of agency skill than person 2. Yet, both individuals behave differently depending on the situation. For example, there are situations in which both show more agentic (i.e., assertive, confident, decisive, and energetic) behavior, situations in which both show less agentic behavior and situations in which person 2 behaves more agentic than person 1.

This understanding of social skills should not be equated with personality traits (e.g., extraversion, agreeableness). Rather, social skills refer to what someone is capable of doing (i.e., when it matters; “maximum performance”), whereas personality traits (in a narrow sense) refer to what someone tends to do in general (i.e., general behavioral tendencies; “typical performance” [2,15,26,34]. A physician who is rather reserved and shy in everyday life but acts assertively when patients are uncooperative should still be considered assertive (in the sense of a skill). Using the example of the broad social skill of agency (i.e., capacity to show assertive, confident, decisive, and energetic behavior), to show the interplay between skills, situations, and behaviors, it becomes clear that, while skills should be considered as cross-situational person characteristics, their specific behavioral expression depends on the situation (Figure 1).

## Taxonomy of Physicians’ Social Skills

### Competency frameworks and analyses of requirements

For a targeted way to measure physicians’ social skills and to establish a shared language between medical school departments, it is necessary to use a taxonomy that allocates the various social skills to overarching and clearly distinguishable skill dimensions. According to the principle of parsimony, as few different dimensions as possible but as many as necessary should be used [35]. Only then will it be possible to identify a clear profile of social skills for medical student selection or development. Competency frameworks, such as the Outcomes for Graduates [36], the Accreditation Council for Graduate Medical Education’s Outcome Project [4], or the CanMEDS framework [3], are only partially suitable for this purpose because the roles or domains described in the frameworks are organized around key content areas, which inevitably overlap significantly. For example, many roles include both specific clinical knowledge elements and social aspects (e.g., in the CanMEDS role of *leader*). At the same time, underlying social skills are relevant to several roles (e.g., relationship building or emotional understanding for *communicator* and *collaborator* roles). This is not a general critique of competency frameworks as the complexity of physicians’ activities makes the overlap inevitable. Also, the frameworks have proven very useful in many areas. Yet, when it comes to a differentiated consideration of physicians’ *social* skills for assessment and training, it is neither sufficient nor practical to consider only the competency roles.

For a more meaningful assessment of social skills, it is therefore necessary to elaborate on the specific skills that underlie the competency roles. There is already some preliminary work on this. For example, Wijnen-Meier and colleagues [37] extracted 25 (cross-situational) skills (*facets of competence*) from various frameworks and studies, most of which can be categorized as social skills (e.g., *teamwork and collegiality, coping with mistakes, responsibility, verbal communication, empathy and openness, active listening, handling emotions, coping with uncertainty*; see also [38,39]). Beyond this research, there are many similar lists of (social) skills that have been based on analyses of requirements or surveys that show the wide range of desirable social skills for physicians (e.g., Hertel-Waszak and colleagues [40]: *communication, teamwork, respect, proactivity, ability to handle stress, ability to handle criticism, moral integrity;* Lambe and colleagues [41]: *compassion, pro-social attitude, communication and listening skills, initiative, probity, ability to cope with ambiguity, ability to be a team player*; Hurwitz and colleagues [42]: *empathy, honesty, ability to work in a team, emotionally stable, decisiveness, leadership abilities*; see also [43,44,45]).

However, such an approach leads to a steadily increasing and hardly manageable number of detached social skills with at times highly overlapping content (e.g., *empathy* and *active listening*). As a result, classic jingle-jangle fallacies can be found. The jingle fallacy describes the false assumption that social skills with the same label also have the same content. An example of this is the skill “*communication*,” which is understood differently depending on the context or the specific individuals using the term. The jangle fallacy describes the false assumption that social skills with different labels actually represent something different. Examples of this fallacy include the skills *empathy, relationship building*, and *social sensitivity*. Whereas these skills have slightly different nuances, at their core, they refer to very similar behavioral expressions between individuals. These issues lead to the problem that when different medical schools or individuals talk about certain social skills, it is often not clear what they really mean. These issues are especially troublesome when it comes to selecting people with specific social skills or for developing certain skills during their studies. Consequently, for a reliable and valid assessment of social skills, it is necessary to first determine in which *distinguishable* social skill dimensions people differ.

### A behavioral-based taxonomy

There have already been attempts to combine different social skills into overarching taxonomies, but the number of relevant dimensions and the level of abstraction vary considerably [2,25,27,40,46,47]. One of the reasons for this variability is that, in many cases, a top-down approach has been followed, which means that skills are extracted and defined on the basis of analyses of requirements or surveys and grouped (i.e., assigned to higher-order skills) according to perceived conceptual proximity. In this article, we want to complement this approach with a behavioral bottom-up approach (i.e., focusing on how individual differences manifest at the behavioral level) which has been used mainly in behavioral personality psychology. As shown earlier, differences in social skills manifest in interpersonal behavior in social situations. Therefore, we want to focus on how these behaviors can be grouped empirically. This approach is an essential complement to the top-down approach because, if theoretically defined skills do not manifest in distinguishable behavior, they cannot be distinctively captured (see [14] for similar reasoning). Complementing existing lists of desirable social skills for physicians with a bottom-up approach of distinguishable overarching behavioral dimensions should reduce jingle-jangle fallacies and lead to a manageable number of skill dimensions for selection and training.

While the structure of interpersonal behavior has not been the focus of previous research on physicians’ skills and competencies, it is a core research topic in behavioral personality psychology [48,49,50,51,52]. Here, a large body of research suggests that the broad spectrum of different interpersonal behaviors is best represented by three distinct behavioral dimensions: agency (i.e., individual differences in getting ahead in social situations), communion (i.e., individual differences in getting along in social situations), and interpersonal resilience (i.e., individual differences in staying calm in social situations).

While the term agency is sometimes understood differently depending on the research tradition, we refer to agency as a core dimension of interpersonal behavior, as described, for example, by Leising and Bleidorn [51] “Agency highlights a person’s motive and capacity to “get ahead” (sometimes ahead of others)” (p. 986). That is, agency includes assertive, confident, determined, and energetic behaviors. These behaviors, in turn, can be attributed to a variety of social skills. For example, individuals who behave dominantly and energetically across a variety of relevant situations demonstrate *assertiveness, decisiveness, responsibility, persuasiveness, or pragmatism*. Communion includes friendly, helpful, and compassionate behaviors and thus combines frequently mentioned social skills such as *cooperativeness, empathy, relationship building, social sensitivity*, or *warmth*. Finally, interpersonal resilience involves dealing with challenging interpersonal situations in a calm, relaxed, and emotionally balanced manner and includes social skills such as the *ability to take criticism, ambiguity tolerance, coping under pressure, emotional control*, or *resistance to stress*.^2^

The division into agency and communion is already part of many definitions of socially skilled individuals (e.g., linking conflict readiness and cooperativeness; [53,54,55]) and is mainly described in the context of research on interpersonal interactions (interpersonal theory; [52,56,57,58,59,60]). The third dimension – resilient behavior – can be derived from a slightly different line of research (interpersonal differences in dealing with stress and uncertainty), is particularly visible in stressful situations and cannot be directly assigned to agency or communion [51,61,62,63,64]. Several empirical studies suggest that these three behavioral dimensions can be measured distinctively and reliably. For example, in an exploratory bottom-up analysis, Leising and Bleidorn [51] were able to show that 35 different (interpersonal) adjectives could be clearly assigned to these three factors and therefore referred to them as “basic meaning dimensions of observable interpersonal behavior”. Stable and distinctively measurable behavioral differences in the three dimensions have also been found in situations relevant to physicians in the context of selecting and training of medical students [10,15,48]. Thus, these behavioral dimensions are not only empirically distinguishable and observable but also relevant in the medical context. Although there are other aspects that are crucial for physicians (e.g., clinical knowledge, analytical thinking, experience), the three dimensions cover almost all important aspects of the interpersonal and social context.

Building on these findings, we recommend defining the three behavioral dimensions (agency, communion, interpersonal resilience) as overarching dimensions of physicians’ social skills. The current approach has several advantages over an intuitive approach to taxonomy building. The three overarching skill dimensions are relevant to the medical context but are also independent of each other because they are based on empirically distinguishable interpersonal dimensions. This independence allows often redundant terms for physicians’ social skills to be combined under three clear labels. For instance, the aforementioned social skills identified by Wijnen-Meier and colleagues [37] can be assigned to the three dimensions (e.g., agency: *responsibility, structure work planning, and priorities;* communion: *teamwork and collegiality, empathy and openness;* interpersonal resilience: *knowing of personal bounds, coping with uncertainty)*. The same applies, for example, to the social aspects of the reference list of general physician competencies by the Association of American Medical Colleges (e.g., agency: *demonstrate self-confidence*; communion: *demonstrate sensitivity, honesty, and compassion*; interpersonal resilience: *demonstrate healthy coping mechanisms to respond to stress* [18]).

Mapping such specific social skills onto overarching skill dimensions in this way not only facilitates cross-context communication but also reduces measurement error based on conceptual ambiguity (for example, it makes it clearer to people who are assessing these skills which behaviors are relevant and which are not). Thus, the three behavior-based dimensions of social skills offer a practically manageable compromise between the more requirement-defined and difficult to distinguish competency roles on the one hand and a multitude of specific skill lists with conceptual ambiguity on the other. Many measurements in the area of social skills can be assigned to this overarching structure, regardless of which measurement level or method of procedure is used (Table 1). Thus, the pooling of data and results not only across sites but also across different measurement points within individual sites can be facilitated (e.g., when tracking student learning progress over time with a wide variety of assessment formats).

**Table 1:** Physicians’ Social Skills: A Behavioral-Based Taxonomy.


PHYSICIANS’ SOCIAL SKILLS		

Entire range of skills (behavioral capacities) that promote effective functioning in interpersonal situations in the medical context (e.g., physician-patient interaction)		

Behavioral-based taxonomy: skill dimensions		

**Agency skill**	**Communion skill**	**Interpersonal resilience**

Physicians with high levels of agency skill recognize the interpersonal situations in which assertive, confident, decisive, and energetic behavior is required and can use such behavior accordingly	Physicians with high levels of communion skill recognize the interpersonal situations in which warm, friendly, and compassionate behavior is required and can use such behavior accordingly	Physicians with high levels of interpersonal resilience recognize the interpersonal situations in which calm, relaxed, and emotionally balanced behavior is required and can use such behavior accordingly

Incorporates skills such as:	Incorporates skills such as:	Incorporates skills such as:

Assertiveness	Cooperativeness	Ability to take criticism

Decisiveness	Empathy	Ambiguity tolerance

Responsibility	Relationship building	Coping under pressure

Persuasiveness	Social sensitivity	Emotional control

Pragmatism	Warmth	Resistance to stress

Characteristics of situations in which behavioral differences become visible		

**Reactance:** Patients who show resistance or need to be convinced **Insecurity:** Patients who show insecurity or need to be carried along and motivated **Decisions:** Situations that require proactive decisions **Conflicts:** Situations in which conflicts need to be addressed (e.g., conveying negative feedback, setting boundaries)	**Need for help:** Patients who are dependent on others or are in pain (e.g., an accident has occurred, suffering patients) **Sadness:** Patients who are distressed or stunned (e.g., physician delivers bad news) **Bad mood:** Communication with patients who are in a bad mood or unhappy	**Social stressors:** Patients who complain or pressure the physician **Performance stressors:** Situations with self-esteem-relevant content **Time pressure:** Situations that require quick decisions (e.g., emergencies) **Uncertainty/ambiguity:** Situations in which there is no clear solution or one is unprepared


## Assessment of Physicians’ Social Skills

In medical education, it has long been discussed whether and how individual differences that go beyond specific clinical knowledge can be measured and quantified. Some authors have argued that many desirable skills are not objectifiable phenomena and therefore may not be measurable at all [14,19,20]. The approach presented here addresses this issue by focusing on reliable and clearly measurable skill dimensions. Considering the three overarching social skill dimensions clarifies why it has often not been possible to measure specific social skills reliably: many social skills manifest themselves in very similar and co-occurring behaviors (e.g., *cooperativeness, empathy, relationship building, social sensitivity, warmth*, all of which revolve around very similar behaviors).

The empirical and theoretical foundation of the three skill dimensions also make it possible to identify the types of situations in which they become visible and measurable. We determined these situation characteristics on the basis of previous research [50,61,62,63,65]. Individual differences in the agency skill dimension become visible in situations involving reactance, decision-making, or conflict. Situations that involve sadness or the need for help primarily evoke individual differences in communion. Finally, stressful situations (e.g., social stressors, performance stressors, time pressure) or ambiguity evoke individual differences in interpersonal resilience (see Table 1 for more specific examples). It can be seen that the measurability of social skills can be ensured through a targeted adaptation of situations, which has implications for the measurement of social skills before and during a student’s studies.

Ideally, measuring a potential student’s social skills before entering medical school (as part of the selection process) is done through behavioral observations in simulated situations. In contrast to other methods, such as classic interviews [8] or situational judgment tests [1,66], not only social knowledge (“How would I behave?”; “knows how” level) is assessed but also the concrete implementation (“How do I actually behave?”; “shows how” level [67,68,69]). We recommend focusing on the three overarching skill dimensions so that students with high levels of proficiency in various skills can be selected (i.e., ideally the selected students should already have a high level in all three skills, meaning they will be able to get ahead in social situations, get along in social situations, *and* stay calm in social situations). Through the targeted use of the situation characteristics, the selection simulations can be designed in such a way that relevant differences in the respective skills can be observed. One way to ensure that only differences in the selected skills are assessed is to develop simulation stations that focus primarily on only one skill dimension at a time (see [15] for a selection procedure that was developed in this way).

When assessing social skills during a student’s course of study, it first is necessary to differentiate between simulated and real situations. Measurements in simulated situations primarily involve simulation patients (e.g., in the form of objective structured clinical examinations [70,71,72]). Again, it is possible to create situations that allow for visible differences in the skill dimensions. As the student’s course of study progresses, clinical content (i.e., taking a patient’s history or taking someone’s blood pressure) can then be added in addition to social requirements. Depending on how the clinical situation is framed, different social skills can be assessed. For example, if the patient whose blood pressure needs to be measured is resistant, differences in agency skill will be visible, and if the patient is sad, differences in communion skill will be visible. This simple adjustment of situations allows targeted training of social skills in a range of relevant clinical contexts. Also, when training social skills, a distinction can be made between the knowledge and implementation levels (e.g., social knowledge: discussing the situations in which it is helpful to behave assertively and confidently; implementation: teaching and practicing assertive rhetoric and body language).

An assessment of social skills in real-life situations takes place, for example, with students in their internship year or in postgraduate training with real patients. In this case, the supervisors usually assess the students with the help of rating instruments. In contrast to simulated situations, a targeted manipulation of the situation characteristics is not possible here. Yet, the division into three overarching social skill dimensions can be well integrated here as well, for example, within the concept of Entrustable Professional Activities (EPA [73,74,75,76,77]). For example, within specific EPAs (e.g., informing and advising patients), recurring situational characteristics that map onto the dimensions of physicians’ social skills can be defined (e.g., communion domain: the student demonstrated compassionate and kind behavior when they had to deliver bad news to patients; interpersonal resilience domain: the student demonstrated calm and relaxed behavior when faced with time pressure when dealing with patients). Such a combination of activities, relevant situation characteristics, and behaviors is much more meaningful than a purely global assessment of specific skills (e.g., the student was empathetic) or activities (e.g., the student was able to advise and inform patients).

Of course, the proposed categorization covers only some of the skills relevant to (future) physicians. For example, the focus on observable social behavior makes it difficult to capture intrapersonal processes (e.g. motivations, ethical values) that may also have an influence on effective functioning in interpersonal situations. Yet, overall, the approach presented could form a common framework for the assessment of observable social skills from the selection process to clinical practice, allowing for flexibility in the measurement of social skills as its use is independent of measurement methods and clinical contexts. Many existing procedures can be classified into this structure by analyzing which behaviors are actually exhibited and which are evaluated in the respective situations.

## Conclusion

A shared understanding of the term “social skills” is needed for effective cross-context communication and for the joint development of procedures to assess physicians’ social skills. In this article, we have moved closer to this goal by proposing a behaviorally and empirically based conceptualization and taxonomy. We see this article as an impetus for a more in-depth discussion of social skills in the medical community and recommend that in the future, the conceptualization, measurement, and communication of physicians’ social skills should be guided by the taxonomy presented.

## Footnotes

In this research, we use the terms “skills” and “competencies” interchangeably. There are studies in which distinctions are made (i.e., competencies as a broader term that incorporates many skills), but differences in which term is used mostly depend on the research tradition (see 25,27). When it comes to the social domains, the term “social skills” is considerably more widespread than the term “social competencies.”

Please note that the “interpersonal resilience” dimension is often also called “emotional stability”. We suggest the label “interpersonal resilience” since it focuses on how one deals with stressors specifically in the interpersonal domain (i.e., staying calm in social situations).
